# Allergic sensitisations during the life course. Results of the KiGGS cohort

**DOI:** 10.17886/RKI-GBE-2018-031

**Published:** 2018-03-15

**Authors:** Roma Thamm, Christina Poethko-Müller, Michael Thamm

**Affiliations:** Robert Koch Institute, Berlin, Department of Epidemiology and Health Monitoring

**Keywords:** IGE ANTIBODIES, CHILDREN AND ADOLESCENTS, COHORT STUDY, HEALTH MONITORING

## Background

Allergic sensitisations of the immune system involve the formation of specific immunoglobulin E (IgE) antibodies after (initial) contact with certain otherwise harmless substances (allergens). Repeated contact with allergens, however, sensitises the immune system. On subsequent contact, the immune system recognises these allergens and this triggers a reaction by its defence mechanisms. Allergic reactions can affect different organs, have different degrees of severity and show various symptoms. Although allergic sensitisations are measurable by analysing the levels of IgE antibodies in the blood, detecting these antibodies does not provide a measurement of disease, rather they are merely associated with an increased risk of allergic diseases [1].

There are four different types of allergic reaction. Type I hypersensitivity, also referred to as the immediate type, is the most common form and it is mediated by IgE antibodies. Some of the best known manifestations of Type I allergies include hay fever and (allergic) asthma. These conditions are among the most common chronic diseases in childhood and adolescence, they place significant burdens on health and have strong socioeconomic consequences [2, 3]. An important aspect of epidemiological allergy research is the extent to which sensitisations persist and how they may develop or even decline during the life course. In particular, this applies to sensitisations to important inhalant allergens that play a significant role in the development of hay fever and asthma. Only limited data are available that can be used to calculate transition probabilities. However, as part of the KiGGS cohort – the largest cohort for children and adolescents in Germany – measurements were taken of important specific IgE antibodies that are associated with the most commonly occurring allergic diseases. These measurements were made during the KiGGS baseline study (2003-2006) and KiGGS Wave 2 (2014-2017). This data can help answer the important question about the extent to which allergic sensitisations persist, arise or even decline over a period of more than ten years. This article, therefore, uses the longitudinal data from the KiGGS cohort to investigate the transition probabilities of allergic sensitisations during the transition from childhood to young adulthood.

## Indicator and methodology

The analyses are based on measurements made of specific IgE antibodies that react against the allergen mixture SX1, a mixture of eight common inhalant allergens (timothy, rye-grass, birch, mugwort, cat and dog dander, house dust mite and the fungus Cladosporium herbarum – Phadia, now Thermo Scientific, Freiburg). Measurements were made from 2,041 girls and 2,143 boys who participated in the cohort study and who were examined both during the baseline study (2003-2006) and during KiGGS Wave 2 (2014-2017). The participants were aged 3 years or older at the time of the first measurement. Transition probabilities were calculated as the percentage probability of a transition from non-sensitisation to sensitisation to the allergen mixture SX1 or vice versa during the period beginning with the KiGGS baseline study and ending with KiGGS Wave 2. The value of ≥0.35 kU/l was used to set the limit of positive sensitisations. A possible bias due to selective re-participation was partially offset by multivariate weighting [4, 5].

**KiGGS Wave 2**

Second follow-up to the German Health Interview and Examination Survey for Children and Adolescents

**Data owner:** Robert Koch Institute

**Aim:** Providing reliable information on health status, health-related behaviour, living conditions, protective and risk factors, and health care among children, adolescents and young adults living in Germany, with the possibility of trend and longitudinal analyses

**Study design**: Combined cross-sectional and cohort study

**Cross-sectional study in KiGGS Wave 2**

**Age range:** 0-17 years

**Population:** Children and adolescents with permanent residence in Germany

**Sampling:** Samples from official residency registries - randomly selected children and adolescents from the 167 cities and municipalities covered by the KiGGS baseline study

**Sample size:** 15,023 participants

**KiGGS cohort study in KiGGS Wave 2**

**Age range:** 10-31 years

**Sampling:** Re-invitation of everyone who took part in the KiGGS baseline study and who was willing to participate in a follow-up

**Sample size:** 10,853 participants

**KiGGS survey waves**

► KiGGS baseline study (2003-2006), examination and interview survey
► KiGGS Wave 1 (2009-2012), interview survey
► KiGGS Wave 2 (2014-2017), examination and interview survey

More information is available at www.kiggs-studie.de/english

## Results

**Figure 1:**
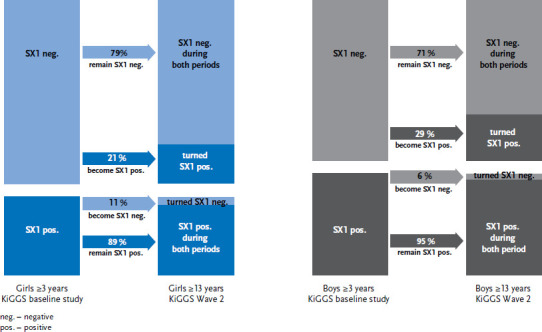
Sensitisation to an allergen mixture of eight common inhalant allergens (SX1 test) over a 10-year period of the life course (n=2,041 girls, n=2,143 boys) Source: KiGGS baseline study (2003-2006), KiGGS Wave 2 (2014-2017)

Data from the KiGGS baseline study show that 30% of girls aged 3 years or older and 39% of boys from the same age group (which is significantly more) were found to be sensitive to at least one of eight major inhalant allergens; in other words, their SX1 test proved positive. Most of these children also continued to have positive SX1 sensitisation a good ten years later (Figure 1). Only a small proportion of girls (11%) and boys (6%) who had shown sensitisation during the baseline study no longer did so during Wave 2. Among the girls and boys who showed no SX1 sensitisation at the time of the KiGGS baseline study, the probability of becoming sensitised was 21% and 29% respectively (a statistically significant difference). This also means that 79% of girls and 71% of boys remained SX1-negative after a good ten years.

## Discussion

This study identified clear positive transition probabilities for sensitisation to a mix of eight major inhalant allergens (SX1 test) for the 10-year follow-up among both genders. As such, a far greater level of SX1 sensitisation developed over the life course than receded. Overall, this development, which was more pronounced among boys than girls, reflects the typical differences in gender and age in the incidence of IgE-mediated allergic diseases. The results underscore the need to further study the factors linked to immune system dysregulation, especially among children with a genetic predisposition to allergies. This would enable relevant preventive and therapeutic measures to be developed.
